# Experimental demonstration of the equivalence of entropic uncertainty with wave-particle duality

**DOI:** 10.1126/sciadv.adr2007

**Published:** 2024-12-06

**Authors:** Daniel Spegel-Lexne, Santiago Gómez, Joakim Argillander, Marcin Pawłowski, Pedro R. Dieguez, Alvaro Alarcón, Guilherme B. Xavier

**Affiliations:** ^1^ Institutionen för Systemteknik, Linköpings Universitet, 581 83 Linköping, Sweden.; ^2^ Departamento de Física, Universidad de Concepción, 160-C Concepción, Chile.; ^3^ Millennium Institute for Research in Optics, Universidad de Concepción, 160-C Concepción, Chile.; ^4^ International Centre for Theory of Quantum Technologies, University of Gdańsk, Jana Bazynskiego 8, 80-309 Gdańsk, Poland.; ^5^ Departamento de Ingeniería Eléctrica y Electrónica, Facultad de Ingeniería, Universidad del Bío-Bío, Avenida Collao 1202, 4051381, Concepción Chile.

## Abstract

Wave-particle duality is one of the most notable and counterintuitive features of quantum mechanics, illustrating that two incompatible observables cannot be measured simultaneously with arbitrary precision. In this work, we experimentally demonstrate the equivalence of wave-particle duality and entropic uncertainty relations using orbital angular momentum (OAM) states of light. Our experiment uses an innovative and reconfigurable platform composed of few-mode optical fibers and photonic lanterns, showcasing the versatility of this technology for quantum information processing. Our results provide fundamental insights into the complementarity principle from an informational perspective, with implications for the broader field of quantum technologies.

## Teaser

The information gain generated from complementary measurements on an individual quantum system is experimentally confirmed.

## INTRODUCTION

The uncertainty principle states that some pairs of observables cannot be known simultaneously with high precision. On the other hand, Bohr’s complementarity principle manifested its key role in modern physics by submitting matter and radiation to a unifying framework, where it is expected to exclusively behave either in a wave- or in a particle-like manner (*1*, *2*) depending on the peculiarities of an interferometric setup (*3*). By examining the operational meaning of min- and max-entropies (*4*, *5*), the uncertainty and complementarity principles were unified for two-path interferometers (*6*). This unification was extended to multipath interferometers in (*7*) by considering wave and particle duality through two complementarity guessing games. This framework introduced a quantifier for fringe visibility applicable to multipath interferometers, revealing a proper trade-off with a generalized version of path distinguishability.

The entropic uncertainty relations (EURs) can be applied to interferometers in two different manners. One concerns preparation uncertainty, where a quantum state cannot be prepared with certainty for two complementary observables, while the other involves measurement uncertainty, indicating that two complementary observables cannot be jointly measured (*6*). Complementarity and uncertainty then pose a challenge to classical assumptions, such as physical realism (*3*, *8*), by proposing that quantum systems may have properties that only emerge once the entire physical context, including the system and the classical measuring apparatus, is lastly settled (*3*, *9*, *10*). Wave-particle duality relations (WPDRs) aim to provide operational meaning to the complementarity principle, as it can be used to serve as the fundamental mechanism driving advantages over classical tasks (*5*).

Several experiments have been conducted to test the robustness of the complementarity principle, particularly through delayed-choice experiments (*11*, *12*) and its generalizations, such as the entanglement-separability duality in bipartite systems (*13*, *14*). This duality is examined in delayed-choice entanglement swapping experiments (*15*) and quantum delayed-choice experiments (*3*, *16*, *17*), which use quantum-controlled gates to create an effective superposition of a present and absent beam splitter (BS) within the experimental setup. Despite these efforts, debates persist (*3*, *12*, *18*–*20*) regarding whether interference phenomena truly defy classical explanations or whether Bohr’s complementarity principle needs updating (*21*).

The complementarity principle plays a key role in various quantum technologies. For example, it governs the relationship between entanglement-assisted and quantum-distributed random access codes (*22*), supports the derivation of security proofs (*23*), and has proven useful in quantum metrology tasks (*24*, *25*). Uncertainty relations are relevant, for instance, in thermodynamics (*26*–*30*), and EURs have shown to be useful in deriving security bounds in quantum cryptography applications (*31*–*34*), including quantum random number generation and quantum key distribution (QKD). The experimental equivalence between EURs and WPDRs may, therefore, have applications across both of these domains, as it opens up alternate methods for assessing relevant quantities.

In this work, we present an experimental demonstration of the equivalence between wave-particle duality and EURs (*6*), for which we rely on states encoded in the orbital angular momentum (OAM) degree of freedom of light (*35*, *36*) to assess the entropic uncertainty both from the definition and using interferometric visibility and input distinguishability (*6*). The OAM of light is widely used in photonic quantum information due to its support for high-dimensional Hilbert spaces (*37*). Traditionally, OAM-based experiments relied on bulk optics and free-space channels (*38*–*45*) due to the challenge of manipulating and transmitting these states over optical fibers, crucial for fiber-optic telecommunication compatibility. Multimode fibers, although capable of supporting OAM modes, require complex compensation systems for stable transmission due to numerous transverse spatial modes (*46*, *47*). Recently, the telecom industry developed spatial division multiplexing (SDM) fibers, which support fewer transverse spatial modes to enhance transmission capacity (*48*), and have become vital for processing and transmitting photonic transverse spatial quantum states (*49*). Few-mode fibers (FMFs), a type of SDM fiber, have successfully transmitted photonic OAM quantum states over long distances (*50*, *51*) by linearly decomposing OAM modes into the linearly polarized (LP) modes supported by the fibers. Furthermore, using photonic lanterns (*52*) in an interferometric setup, we can dynamically excite different OAM modes in an FMF with ultrafast response times (*53*). This method also enables ultrafast reconfigurable projective measurements on OAM states (*54*). Using these techniques, we perform here entropic uncertainty measurements on the wave and particle aspects of OAM states using an FMF interferometric setup combined with a fiber-optical tunable BS based on a Sagnac interferometer (SI). Our setup can dynamically change the measurement operator such that the two complementarity extremes can be measured, as well as any value in between. We are able to experimentally verify the entropic uncertainty relations revealing the operational meaning of the complementarity principle, as well as opening more applications for quantum information processing.

## RESULTS

### Preliminaries: EURs and WPDRs

Wave and particle behaviors are quantified by the knowledge related to mutually unbiased observables (*5*–*7*) within the entropic uncertainty framework (*6*). The related WPDR relies on the operational connection between the min- and max-entropies, with guessing probabilities. The wave-particle duality emerges in this context as a fundamental bound for two complementarity guessing games (*5*). In the following, we briefly introduce the framework developed in (*6*, *7*), to present our experimental confirmation of the WPDR and EUR equivalence. Consider a classical-quantum state $\Phi_{A}\left(\rho_{AB}\right)≔\sum_{a}\;p_{a}|a\rangle \langle a|⊗\rho_{B}^{a}$ in a Hilbert space ${H_{A}}^{\left(n\right)}⊗{H_{B}}^{\left(m\right)}$ such that n and m are the dimensions of each subspace, and $\rho_{B}^{a}=\langle a|\rho_{AB}|a\rangle /p_{a}$ with $p_{a}=\text{Tr}\left[\left(|a\rangle \langle a|⊗1\right)\rho_{AB}\right]$. Setting Z as the which-path random variable associated with the basis ℤ={∣z〉〈z∣} of an n-dimensional Hilbert space $H_{A}$, and W as the random variable associated with a mutually unbiased basis W in respect to ℤ, the following optimized wave and particle EUR

$$H_{\text{min}}\left(Z|B\right)+\underset{W}{\text{min}}H_{\text{max}}\left(W\right)\geq \text{lo}g_{2}n$$ (1)

states that for a n-path interferometer, the sum of the ignorance about wave and particle behaviors is at least $\text{lo}g_{2}n$ bits of information (*5*). In Materials and Methods, we detail the framework developed in (*6*, *7*) that shows the equivalence between the EUR described in Eq. 1 with a generalized path distinguishability and interferometric visibility such that $D^{2}+V^{2}\leq 1$ holds (*7*).

**Fig. 1.**
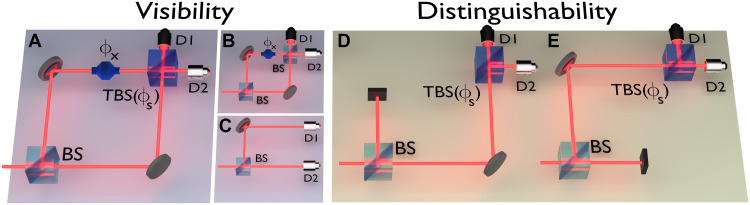
Different experimental configurations for complementarity measurements. (**A**) MZ interferometer with a TBS to recombine the paths and a modulator applying a relative phase shift $\phi_{x}$ between the two arms. (**B**) TBS adjusted to equal transmission and reflection coefficients, yielding full interferometric visibility. (**C**) TBS adjusted to complete transmission or reflection, equivalent to it being removed. In this case, full path information is available; thus, no interference can be observed. (**D** and **E**) For the distinguishability measurements, each path is individually blocked, and the detection events are recorded for any setting of the TBS. In both cases, no interference pattern is possible.

For binary interferometers, particle behavior is quantified by the knowledge of the observable Z which indicates the path the system has taken inside the interferometer. In contrast, wave properties are often modeled as having a well-defined phase and being spatially delocalized. Hence, for a two-dimensional Hilbert space $H_{A}$, wave properties are associated with the eigenstates of observables living in the X−Y plane of the Bloch sphere, describing a set of observables that are mutually unbiased with Z. Here, we use an interferometric setting for which we will first refer to a standard Mach-Zehnder (MZ) interferometer where the final BS, responsible for the recombination of the wave packets, is replaced with a tunable beam splitter (TBS) (Fig. 1), to test the equivalence of wave-particle duality with the following EUR

$$H_{\text{min}}\left(Z\right)+\underset{W}{\text{min}}H_{\text{max}}\left(W\right)\geq 1$$ (2)

The equivalence with a WPDR in our interferometric setting depicted in Fig. 1, which uses a 50:50 BS at the input, is obtained by demonstrating that min- and max-entropies are respectively related to particle and wave information of a quantum state as (*6*)

$$H_{\text{min}}\left(Z\right)=-\text{lo}g_{2}\frac{1+D}{2}$$ (3)

where the input distinguishability D can be experimentally obtained as the average

$$D≔\frac{1}{2}\left(D_{1}+D_{2}\right)$$ (4)

such that

$$D_{1\left(2\right)}≔{\left[\frac{|p_{1}-p_{2}|}{p_{1}+p_{2}}\right]}_{\text{path}1\left(2\right)\text{blocked}}$$ (5)

and

$$\underset{W}{\text{min}}H_{\text{max}}\left(W\right)=\text{lo}g_{2}\left(1+\sqrt{1-V^{2}}\right)$$ (6)

with the usual interferometric visibility for a two-path interferometer defined as

$$V≔\frac{p_{j}^{\text{max}}-p_{j}^{\text{min}}}{p_{j}^{\text{max}}+p_{j}^{\text{min}}}$$ (7)

where the maximization and minimization over the detection probability $p_{j}$ with j=1,2 are performed under the controllable phase of the interferometer $\phi_{x}$ (as depicted in Fig. 1). In this interferometric arrangement, a single photon interacts with a BS generating a path superposition state across the two paths, with a relative phase applied to one of the paths, creating the state $|\psi \rangle =\frac{1}{\sqrt{2}}\left(|0\rangle +{\mathrm{ie}}^{i\phi_{x}}|1\rangle \right)$, where ∣0〉 and ∣1〉 correspond to a path at the output of the 50:50 BS respectively, and the paths are recombined at a TBS (Fig. 1A). The relative phase $\phi_{x}$ is added by a phase modulator, with *i* being the imaginary component.

A TBS has the capability of dynamically changing its transmission (t) and reflection coefficients (r), obeying the condition that ${|r|}^{2}+{|t|}^{2}=1$. The interferometer has two orthogonal outputs where single-photon detectors D1 and D2 are placed. If the two paths are indistinguishable, then the probability amplitudes interfere at the second BS, with full interferometric visibility achieved if the TBS is set such that r=t equivalent to a standard 50:50 BS (Fig. 1B). On the other hand, if the BS is completely removed (t or r=1), then we have the situation in Fig. 1C, in which the paths are completely distinguishable, and no interference behavior can be observed. It is also possible to obtain partial wave and particle behavior, effectively going through a continuum between the two extrema cases by adjusting the TBS.

The visibility can be measured directly for different settings of the TBS from the detection probabilities at the outputs of the interferometer. Last, to quantify the distinguishability, each path needs to be individually blocked (Fig. 1, D and E), and thus, the probabilities can be measured for any state of the TBS.

### Experimental description

**Fig. 2.**
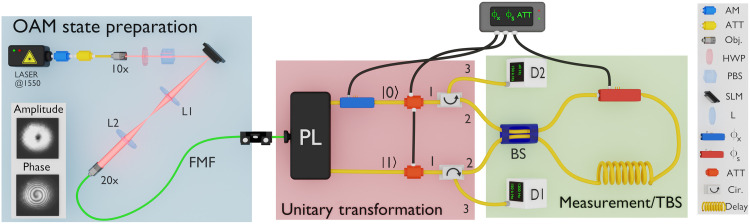
Experimental setup for probing the EUR over a |OAM_+1_〉 qubit. The experiment consists of three main parts: the $|{\text{OAM}}_{+1}\rangle$ mode source, the unitary transformation, and the measurement stage (or TBS). An amplitude modulator (AM) and an attenuator (ATT) adjust the average number of photons per pulse to μ=0.2. In addition, the $|{\text{OAM}}_{+1}\rangle$ state can be encoded by applying the appropriate forked diffraction grating to the spatial light modulator (SLM) and also using a 4f system (see Materials and Methods). The amplitude and phase profiles of the ${\text{OAM}}_{+1}$ state are shown, following propagation through the FMF with an InGaAs infrared camera with the laser source unattenuated. Then, the unitary transformation stage performs a mapping from spatial to path information, yielding $|\psi \rangle =\frac{1}{\sqrt{2}}\left(|0\rangle +e^{i\phi_{x}}|1\rangle \right)$, where a rotation to the state can be applied through the phase modulator $\phi_{x}$. The TBS is described by an SI containing an optical delay and a phase modulator $\phi_{s}$, which controls the transmissivity and reflectivity of the BS, which determines the maximum obtained visibility. On the other hand, to measure the distinguishabilities $D_{1}$ and $D_{2}$, it is necessary to block the paths before the SI with electro-optical ATTs.

On the basis of an all-in-fiber interferometer, we have implemented an experimental setup to test the EUR on an OAM quantum state. Instead of using a standard MZ interferometer, our setup is innovatively designed to observe the wave or particle behavior of an OAM state using SDM devices and an SI as a TBS. The experimental setup, shown in Fig. 2, can be divided into three main stages: state preparation, unitary transformation, and measurement (or tunable BS). Let us present these three stages in sequence.

A weak coherent state (WCS) is prepared using a continuous-wave telecom laser operating at 1550 nm connected to a fiber-pigtailed amplitude modulator generating a pulse width of 40 ns at a repetition rate of 150 kHz, in series with an optical attenuator (ATT) to bring down the optical power to a single-photon level. Next, a spatial light modulator (SLM) and a 4f system are used to prepare the $|{\text{OAM}}_{+1}\rangle$ state (see Materials and Methods). The light is coupled into an FMF capable of supporting three spatial modes, the fundamental LP mode ${\text{LP}}_{01}$ and the two degenerate higher-order modes ${\text{LP}}_{11a}$ and ${\text{LP}}_{11b}$. Within the FMF, the OAM state is decomposed into LP components as follows $|{\text{OAM}}_{+1}\rangle =\frac{1}{\sqrt{2}}(|{\text{LP}}_{11a}\rangle +i|{\text{LP}}_{11b}\rangle$ (*51*), where i is the relative phase between the LP modes. We image the ${\text{OAM}}_{+1}$ mode after coupling to the FMF, by imaging its facet onto an InGaAs (Indium Gallium Arsenide) charge-coupled device infrared camera. The amplitude profile is imaged directly, while for the phase profile, we interfere the OAM state with a Gaussian beam generated by the same laser and split before the SLM (camera setup not shown for simplicity) (*53*). This measurement is done with the laser without attenuation and in continuous wave mode and the results shown in the inset of the OAM state preparation stage in Fig. 2.

When the OAM state reaches the unitary transformation stage, a three-mode photonic lantern (Phoenix Photonics) is used as a spatial demultiplexer to perform a mapping from modal to path information as follows: $|{\text{LP}}_{11a}\rangle \to |0\rangle$ and $|{\text{LP}}_{11b}\rangle \to |1\rangle$, where ∣0〉 and ∣1〉 are the upper and lower arms of the interferometer, respectively. The third port of the lantern, corresponding to the fundamental ${\text{LP}}_{01}$ mode, is not used. The two paths following the lantern mapping operation are single-mode optical fibers. A lithium niobate (LiNbO_3_) fiber-pigtailed telecom phase modulator $\left(\phi_{x}\right)$ is placed in the upper path, allowing unitary transformations on the superposition state $\frac{1}{\sqrt{2}}\left(|0\rangle +e^{i\phi_{x}}|1\rangle \right)$. Manual polarization controllers (not shown for the sake of simplicity) are placed in each arm to optimize interference in the 50:50 fiber BS, as well as two variable electro-optical attenuators (Thorlabs, V1550A) to control the transmissivity of each path.

In transitioning from the unitary transformation stage to the measurement stage, each of the two paths passes through two optical circulators, which transmit each path component forward (1→2; see Fig. 2) to a 50:50 fiber BS, forming a fiber-optical SI. The SI operates as a tunable BS controlled by a LiNbO_3_ phase modulator $\left(\phi_{s}\right)$. Following the BS, two wave packets are generated within the interferometer, traveling clockwise and counterclockwise. A 300-m fiber optic delay line is used to ensure sufficient time separation for the phase modulation signal $\left(\phi_{s}\right)$ to act on the wave packet propagating in only one of the internal directions, thus creating the change of relative phase necessary to generate tunability on the SI outputs (*55*). Within the SI, there are two manual polarization controllers (also not shown for simplicity) to ensure that both wave packets recombine with the appropriate polarization in the BS, as well as aligning the polarization state at the phase modulator. After recombination, the wave packets are sent again to the circulators (now taking the direction 2→3) and are detected at D1 and D2. We use one single-photon detector with a time multiplexing scheme to be able to measure both outputs simultaneously (Materials and Methods). The detector is an InGaAs-based single-photon counting module (ID Quantique id210) running in gated-mode with 10% overall detection efficiency, 3-ns-wide gate windows. Both the $\phi_{s}$ modulator and the detector are synchronized with the repetition rate of the source. The total loss of the measurement system is approximately 12 dB, including the optical circulators. We adjust the input optical attenuator at the beginning of the setup to obtain 0.2 photons on average per detection gate of 3 ns just after the state is prepared by the unitary transformation operation. As such, a multiphoton probability of less than 2% is achieved. Although similar results could be obtained with a laser operating in the classical regime, the use of weak coherent pulses opens the possibility of expanding our implementation toward QKD protocols (*56*) that are based on delayed choice complementary measurements (*57*) as well as other fundamental tests using prepare-and-measure protocols (*58*).

When $\phi_{s}=0$, the SI behaves like a mirror, essentially equivalent to having no BS installed at the measurement stage, as both inputs to the BS are reflected to the corresponding circulators. Despite continuously modulating $\phi_{x}$, no interference pattern (particle behavior) occurs, as deduced from Eqs. 23 and 24 in Materials and Methods. On the other hand, when $\phi_{s}=\pi /2$, the SI operates as a BS with equal transmission and reflection coefficients. Consequently, the measurement will exhibit interference fringes with maximum contrast (wave behavior). Any intermediate $\phi_{s}$ value between 0 and π/2 will result in the generation of a partial interference pattern (see Materials and Methods for more details). Last, the distinguishability can be assessed by blocking one of the paths in the unitary transformation stage using the variable electro-optical attenuators.

**Fig. 3.**
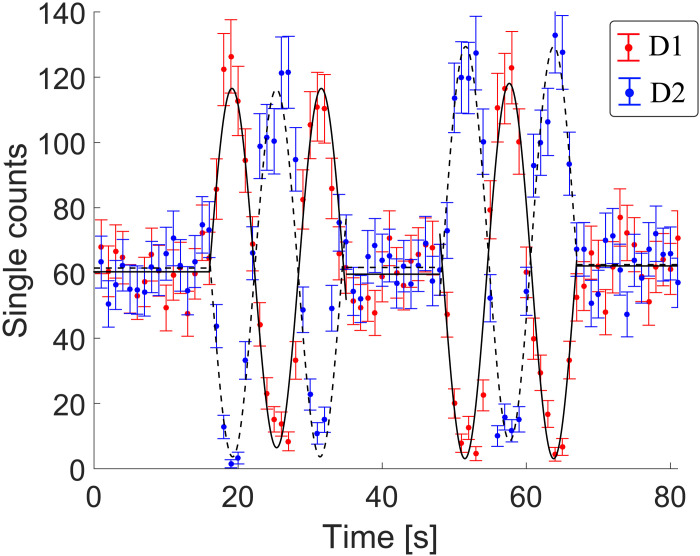
Dynamic change between OAM wave and particle behavior. Recorded detections at D1 and D2 with a continuous triangle waveform applied to $\phi_{x}$ and $\phi_{s}$ periodically changed between 0 (particle) or π/2 (wave). Error bars are the SD considering Poissonian statistics of the single-photon detection process.

An important feature of our experimental setup is the reconfigurability of the measurement from mirror to BS mode due to the use of a fast electro-optical modulator controlling $\phi_{s}$. We demonstrate this dynamic reconfigurability by continuously modulating $\phi_{x}$ with a triangular waveform and switching $\phi_{s}$ from 0 to π/2 every 18 s approximately. These results can be observed in Fig. 3, where we see the switching from particle to wave behavior owing to the state of the TBS.

**Fig. 4.**
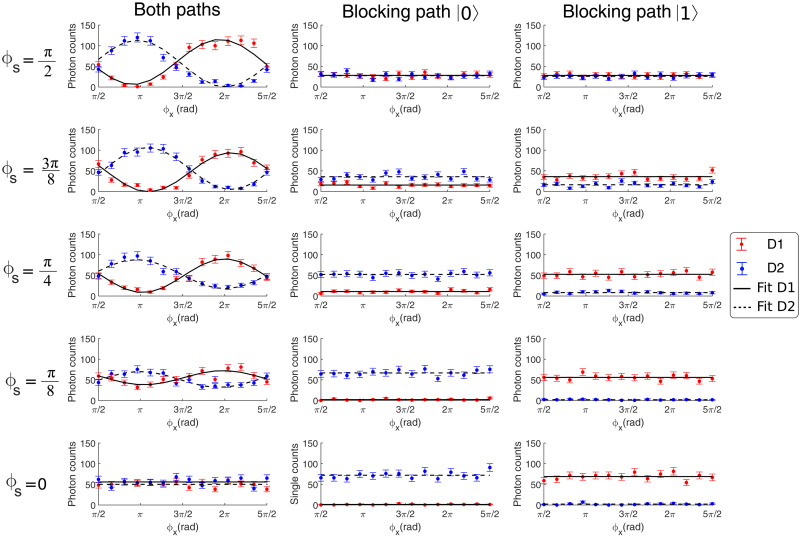
Single counts for detectors 1 and 2 as a function of $\phi_{x}$, varying the phase shift $\phi_{s}$ applied in the SI. In the first column, both arms of the interferometer are open, while the second and third columns show the single count when paths 0 or 1 are blocked, respectively. In each subplot, the error bars were calculated through error propagation taking into account the Poissonian statistics of the recorded individual counts. The integration time for each point is 0.8 s. Last, the solid and dashed lines represent the best-fit curves obtained by minimizing the mean square error between the experimental data and the fit.

To experimentally verify the equivalence between wave-particle duality and EUR, we measure the interferometric visibility and distinguishability of the OAM state for different TBS configurations to evaluate the entropies, and we compare these measurements with the entropies being directly obtained from the probabilities (see Materials and Methods for details). We adjust the phase parameter $\phi_{s}$ to nine different values during these measurements while continuously varying $\phi_{x}$. We show five different configurations of the TBS in Fig. 4, including the two extreme configurations $\phi_{s}=\pi /2$ (BS mode) and $\phi_{s}=0$ (mirror mode). The first column is when both paths are open. The second (third) column is when the variable attenuator blocks the ∣0〉 (∣1〉) path. Each plot shows the number of counts from D1 and D2 versus $\phi_{x}$. In the case where both paths are open, the maximum visibility is 96.7±2.3%. As expected, no interference pattern is observed when $\phi_{s}=0$. In addition, when one of the paths is blocked, no interference pattern is visible. However, a difference appears when the TBS is continuously switched from full BS to mirror mode when one of the paths is blocked. When $\phi_{s}=\pi /2$, the SI acts as a 50:50 BS, and both the detectors measure the same number of counts. As $\phi_{s}$ goes to 0, the detection probability at one of the detectors gradually increases until all the counts are obtained in one detector at $\phi_{s}=0$.

From the probabilities depicted in Fig. 4, the visibility (Eq. 7) and distinguishability (Eq. 4) are calculated for different TBS configurations, which are then used to calculate the min- and max-entropies according to Eqs. 3 and 6. From these two quantities, the EUR (Eq. 2) is first obtained. To show the experimental equivalence, we also use the final interferometric probabilities directly into the definitions of the unconditional min- and max-entropies respectively defined for a given probability distribution $P=\left\{p_{j}\right\}$ as (see Materials and Methods for more details)

$$H_{\text{max}}\left(P\right)≔2\text{lo}g_{2}\sum_{j}\sqrt{p_{j}}$$ (8)

and

$$H_{\text{min}}\left(P\right)≔-\text{lo}g_{2}\underset{j}{\text{max}}p_{j}$$ (9)

**Fig. 5.**
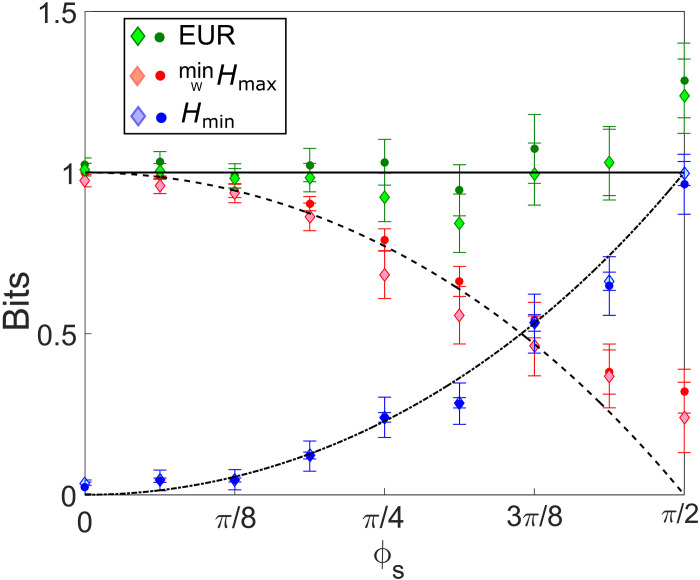
Experimental equivalence between WPDR and EUR. The solid, dashed, and dot-dashed curves are respectively the theoretical values, as a function of the Sagnac phase $\phi_{s}$, of the EUR, minimized max-entropy related to the random variable W (wave), and min-entropy related to the random variable Z (particle). The black, blue, and red circles with their error bars are obtained, for each value of $\phi_{s}$, by measuring the input distinguishability D and the interferometric visibility V, to evaluate the entropies via Eqs. 3 and 6. The black, blue, and red diamonds with their error bars are obtained by taking the measured probabilities related to each variable and applying the entropic definitions as given by Eqs. 8 and 9. Error bars were calculated using error propagation assuming Poissonian statistics for the recorded number of detection events.

Figure 5 presents our main result: The WPDR based on input distinguishability and interferometric visibility is experimentally equivalent to the optimized EUR based on the unconditional min- and max-entropies. The discrepancy observed in the last measurement is due to the limited reach in the visibility of our setup, mainly given by modal cross-talk in the photonic lantern. Because of the squared dependence with the visibility, one needs a very high visibility to be very close to an ignorance of 0 bits for this measurement at an angle $\phi_{s}=\pi /2$. The same is not observed for the distinguishability since there is no square dependence in this case. In the Supplementary Materials (fig. S1), we also plot the wave-particle duality $D^{2}+V^{2}\leq 1$ (*1*, *2*) as a function of the TBS’s setting, thus showing that our experimental setup is also directly applicable to standard complementarity tests.

## DISCUSSION

Wave-particle duality is a well-known hallmark feature of quantum mechanics, and it limits the type of measurements one can do on a quantum system simultaneously. In (*6*), a theoretical framework was established to prove the equivalence between WPDRs and EURs. In this work, we experimentally verified the equivalence between the WPDR based on the input distinguishability D and interferometric visibility V, with the optimized EUR based on the two random variables Z (associated with particle behavior) and W (associated with wave behavior). Our results corroborate the predictions and, once more, confirm Bohr’s complementarity principle which states that individual quantum systems carry information that cannot be fully extracted from the same experimental arrangement. As shown in our main result (Fig. 5), regardless of the interferometer’s configuration, there is always at least one bit of ignorance regarding the information associated with the random variables Z and W when measuring D and V directly. Moreover, by comparing the two different ways of assessing the EUR, Fig. 5 shows that, up to error bars, both quantities are the same. Beyond its foundational implications, these results are also highly relevant from a quantum information perspective as most quantum communication protocols rely on measurements with incompatible operators, thus providing another perspective for practical applications based on the operational meaning of the min- and max-entropies (*5*).

We also have demonstrated a technique to process OAM quantum states of light, based on FMFs, a photonic lantern, and an SI operating as a tunable BS. The wave-particle duality measurement of OAM states in an all-fiber platform opens alternative possibilities to process OAM states with fast response times, due to the electro-optical modulators used, a sharp contrast to the more usual bulk optic components such as q-plates and SLMs. Furthermore, the use of FMFs to transport spatial photonic qubits has shown excellent stability (*51*) over practical distances, further increasing practical applications for the platform shown here. Last, the fast response times of our setup can be used to expand this experiment to other applications such as delayed-choice quantum communication (*57*) using OAM or path-encoded quantum states, giving further alternatives for quantum information protocols. 

## MATERIALS AND METHODS

### EUR and WPDRs in interferometric settings

Here, we define the relevant quantities and briefly explain the theoretical framework based on the operational meaning of the max- and min-entropies and their connection with wave-particle duality (*6*, *7*). For a classical-quantum state $\Phi_{A}\left(\rho_{AB}\right)=\sum_{a}\;p_{a}|a\rangle \langle a|⊗\rho_{B}^{a}$ in a Hilbert space ${H_{A}}^{\left(n\right)}⊗{H_{B}}^{\left(m\right)}$, the min-entropy is defined as (*4*)

$$H_{\text{min}}\left(A|B\right)≔-\text{lo}g_{2}p_{\text{guess}}\left(A|B\right)$$ (10)

where

$$p_{\text{guess}}\left(A|B\right)≔\underset{M_{a}}{\text{max}}\sum_{a}\;p_{a}\text{Tr}\left[M_{a}\rho_{B}^{a}\right]$$ (11)

is the probability of guessing A correctly given the outcome of the optimal positive operator-valued measure (POVM) measurement $M_{a}$ on system B. As discussed before, the entropic formulation associates a kind of behavior (wave and particle) with the availability of a kind of information, or lack of information of the associated random variable (*6*), which means that here A is either the wave random variable W or the particle Z. Moreover, it is important to mention that subsystem B can represent any other internal degree of freedom, such as polarization, or any external environmental system that might interact and encode information about the random variables. Note that, if $\rho_{AB}$ is a product state, this means that subsystem B is considered trivial and $\rho_{A}=\sum_{a}\;p_{a}|a\rangle \langle a|$. In this case, the unconditional min-entropy for a given probability distribution $P=\left\{p_{j}\right\}$ can be computed as

$$H_{\text{min}}\left(P\right)≔-\text{lo}g_{2}\underset{j}{\text{max}}p_{j}$$ (12)

The max-entropy is defined as

$$H_{\text{max}}\left(A|B\right)≔\text{lo}g_{2}p_{\text{secr}}\left(A|B\right)$$ (13)

where

$$p_{\text{secr}}\left(A|B\right)≔\underset{\sigma_{B}}{\text{max}}F\left(\rho_{AB},1⊗\sigma_{B}\right)$$ (14)

quantifies the secrecy of A from B, as measured by the maximum possible fidelity of $\rho_{\mathrm{AB}}$ to an uncorrelated state $\sigma_{B}$ (*7*). Moreover, the unconditional max-entropy is defined as

$$H_{\text{max}}\left(P\right)≔2\text{lo}g_{2}\sum_{j}\sqrt{p_{j}}$$ (15)

For more details regarding applications such as how the min-entropy is used in QKD to quantify how well the eavesdropper can guess the secret key, we refer the reader to (*5*).

Equation 1 can be proved as a WPDR by examining the connection between the max-entropy and guessing probability by the upper bound introduced in (*7*)

$$H_{\text{max}}\left(A|B\right)\leq \text{lo}g_{2}\left\{1+\sqrt{{\left(n-1\right)}^{2}-{\left[{\mathrm{np}}_{\text{guess}}\left(A|B\right)-1\right]}^{2}}\right\}$$ (16)

and noting that minimizing $\text{mi}n_{W}H_{\text{max}}\left(W\right)$ means maximizing $p_{\text{guess}}\left(W\right)$ over all possible basis W. The equivalence of EUR and WPDR can then be demonstrated according to some particular interferometric setting. For instance, when we have a symmetric superposition of paths inside a multipath interferometer, Eq. 1 translates to

$$D^{2}\left(Z|B\right)+V^{2}\leq 1$$ (17)

where

$$V≔\frac{{\mathrm{np}}_{\text{guess}}^{\text{ma}x_{\left\{\phi_{k}\right\}}}\left(W\right)-1}{n-1}$$ (18)

is the generalized notion of interferometric visibility related to the set of relative controllable phases $\left\{\phi_{k}\right\}$, which exhibits a trade-off with the generalized version of path distinguishability

$$D\left(Z|B\right)≔\frac{{\mathrm{np}}_{\text{guess}}\left(Z|B\right)-1}{n-1}$$ (19)

For binary interferometers and treating the second subsystem as trivial as stated in the EUR Eq. 2, Eqs. 18 and 19 can be experimentally obtained as respectively described in Eqs. 4 and 7 and depicted in Fig. 1. In the following, we detail their theoretical description using the states of the interferometer depicted in Fig. 2. For this, we will use the well-known operators that allow mapping some optical components into their matrix representations. Note that, as depicted in Fig. 2, when the single photon enters the SI through BS2, its probability amplitudes recombine in BS2 itself after traveling the entire interferometer. This occurs both in the clockwise and counterclockwise directions; therefore, when producing interference at the output of the SI, a $\frac{\pi }{2}$-phase difference is considered. So, it follows that each one is modeled by

$$\text{BS}1=\frac{1}{\sqrt{2}}\left(\begin{array}{cc} 1 & i\\ i & 1 \end{array}\right),\text{BS}2=\frac{1}{\sqrt{2}}\left(\begin{array}{cc} i & -1\\ -1 & i \end{array}\right)$$ (20)

Two quantum operators corresponding to both phase modulators are presented below

$$\text{PM}1=\left(\begin{array}{cc} 1 & 0\\ 0 & e^{i\phi_{x}} \end{array}\right),\text{PM}2=\left(\begin{array}{cc} 1 & 0\\ 0 & e^{i\phi_{S}} \end{array}\right)$$ (21)

The interferometric output state can be written as

$$\begin{array}{c} |\psi \rangle =\frac{1}{2\sqrt{2}}\begin{array}{c} \left\{i\left[\left(1+e^{i\phi_{s}}\right)+e^{i\phi_{x}}\left(1-e^{i\phi_{s}}\right)\right]|D_{1}\rangle \right.\\ \left.+\left[\left(e^{i\phi_{s}}-1\right)-e^{i\phi_{x}}\left(1+e^{i\phi_{s}}\right)\right]|D_{2}\rangle \right\} \end{array} \end{array}$$ (22)

The probabilities of detecting photons in $D_{1}$ and $D_{2}$ are

$$p_{1}≔{|\langle D_{1}||\psi \rangle |}^{2}=\frac{2-\text{cos}\left(\phi_{x}+\phi_{s}\right)+\text{cos}\left(\phi_{x}-\phi_{s}\right)}{4}$$ (23)

and

$$p_{2}≔{|\langle D_{2}||\psi \rangle |}^{2}=\frac{2+\text{cos}\left(\phi_{x}+\phi_{s}\right)-\text{cos}\left(\phi_{x}-\phi_{s}\right)}{4}$$ (24)

From here, we can derive the expressions for the max-entropy by applying it to the state inside of the interferometer, it reads

$$\begin{array}{c} \underset{W}{\text{min}}H_{\text{max}}\left(W\right)=\underset{\phi_{x}}{\text{min}}\;\text{lo}g_{2}\left(1+\sqrt{1-\text{sin}{\phi_{s}}^{2}\text{sin}{\phi_{x}}^{2}}\right)\\ =\text{lo}g_{2}\left(1+\sqrt{1-\text{sin}{\phi_{s}}^{2}}\right) \end{array}$$ (25)

where $V=\text{sin}\phi_{s}$. On the other hand, distinguishability is obtained by either blocking the lower or the upper path in the first stage of our interferometer. For instance, by blocking the upper path of the state inside the interferometer, the output state reads

$$|\psi \rangle =\frac{1}{2}\left[i\left(1+e^{i\phi_{s}}\right)|D_{1}\rangle +\left(1-e^{i\phi_{s}}\right)|D_{2}\rangle \right]$$ (26)

We can observe that this equation is equivalent to that of a traditional SI when only one input is connected, which is an expected outcome when one of the paths of the interferometer is blocked, as depicted in Fig. 1 (D and E). Furthermore, the wave function does not depend on $\phi_{x}$, as this relative phase arises only when both arms are connected. One can find the input distinguishability using the state inside the interferometer as

$$H_{\text{min}}\left(Z\right)=-\text{lo}g_{2}{\text{cos}}^{2}\left(\phi_{s}/2\right)=-\text{lo}g_{2}\left(\frac{1+\text{cos}\phi_{s}}{2}\right)$$ (27)

such that $D=\text{cos}\phi_{s}$. The above relations for max- and min-entropies as a function of V and D prove the equivalence of Eq. 2 with

$$D^{2}+V^{2}\leq 1$$ (28)

### OAM state preparation

To prepare the OAM state, it is necessary to create a helical mode. This is achieved using a computer-generated hologram displayed on an SLM, which functions as an optical device capable of converting a Gaussian mode into an OAM mode. For optimal operation of the SLM, a Gaussian beam must be propagated, and the laser’s polarization must be set to horizontal. The Gaussian profile naturally comes from the single-mode fiber connected to the laser, while a polarizing beam splitter (PBS) in conjunction with a half-wave plate in free space ensures horizontal polarization before the SLM (Fig. 2). In addition, the beam is collimated using a 10× objective, resulting in a beam waist of approximately 1250 μm, which is sufficient to fully illuminate the forked hologram. The forked hologram consists of a helical phase profile superposed with a linear phase ramp to isolate the encoded field from the Gaussian mode, resulting in a diffraction grating that produces the ${\text{OAM}}_{+1}$ mode in the first diffraction order (*54*). To select the first order of the diffraction, a 4f system is used. This system is composed of two lenses, L1 and L2, each with a focal length of 150 mm. The Fourier transform of the reflected field is located in the focal plane of L1, where a pinhole is placed to pick out the first-order diffraction, which corresponds to the Fourier spectrum of the encoded field. Then, the second lens L2 performs an inverse Fourier transform to lastly obtain the ${\text{OAM}}_{+1}$ mode. A 20× objective is used to couple the ${\text{OAM}}_{+1}$ mode into an FMF, which is placed at the image plane of the 20× objective lens. This procedure allows the generation of WCSs carrying ${\text{OAM}}_{+1}$, which are then propagated through the FMF.

### Single detector scheme

We used one single-photon detection counting module to measure the detection probabilities at both outputs of our setup. To be able to simultaneously measure both outputs with a single detector, we use a time-multiplexing scheme (*55*). A relative optical fiber delay of 1250 ns is inserted at the D1 output in Fig. 1, which is then connected to a fiber-optical PBS, with the other input to the PBS connected to the D2 output. Manual polarization controllers are used to optimize the transmission of both PBS inputs to the same common output, which is then connected to the single-photon detector. Depending on the arrival time (early or late), the processing electronics assign the corresponding output to the detection event. Therefore, for each created WCS, the single-photon detector is gated twice, one for the early and the other for the late detection event. The delay between the two consecutive gates is sufficiently large to not have a substantial after-pulse probability in the late gate. This technique places an upper bound on the repetition rate of the experiment. This scheme is nevertheless also useful in many QKD schemes, as it prevents some side-channel attacks that depend on the physical asymmetries between two single-photon detectors (*59*).
