# Supplementary material for: Experimental demonstration of the equivalence of entropic uncertainty with wave-particle duality
Source: Sci Adv. 2024 Dec 6;10(49):eadr2007. doi: 10.1126/sciadv.adr2007 (PMC11623295; doi:10.1126/sciadv.adr2007)
Supplement: Supplementary file 1 — Supplementary Text Fig. S1 [file sciadv.adr2007_sm.pdf]

Science Advances
AAAS

Supplementary Materials for

Experimental demonstration of the equivalence of entropic uncertainty with wave-particle duality

Daniel Spegel-Lexne *et al.*

Corresponding author: Guilherme B. Xavier, guilherme.b.xavier@liu.se

Sci. Adv. **10**, eadr2007 (2024)
DOI: 10.1126/sciadv.adr2007

This PDF file includes:

Supplementary Text
Fig. S1

Wave-particle duality

The experimental equivalence of the entropic uncertainty relations and wave-particle duality based on input distinguishability and interferometric visibility is demonstrated in our main result shown in Fig. 5 in the main text. In this Supplemental Material, we test the well-known wave-particle inequality for single systems in a two-path interferometer in which the relation needs to obey the following bound (1,2):

$$\mathcal{D}^2 + \mathcal{V}^2 \leq 1 \tag{S1}$$

where $\mathcal{D}$ and $\mathcal{V}$ correspond to distinguishability and visibility as defined in the main text. We verified the inequality by taking the corresponding data for the two variables from the experimental results displayed in Fig. 4 of the main text for the different settings of the tunable beamsplitter and we plot these in Fig. S1, showing the inequality is obeyed. Therefore, we confirm the functioning of our experimental setup to test the complementarity behavior of OAM quantum states in an all-fiber configuration.

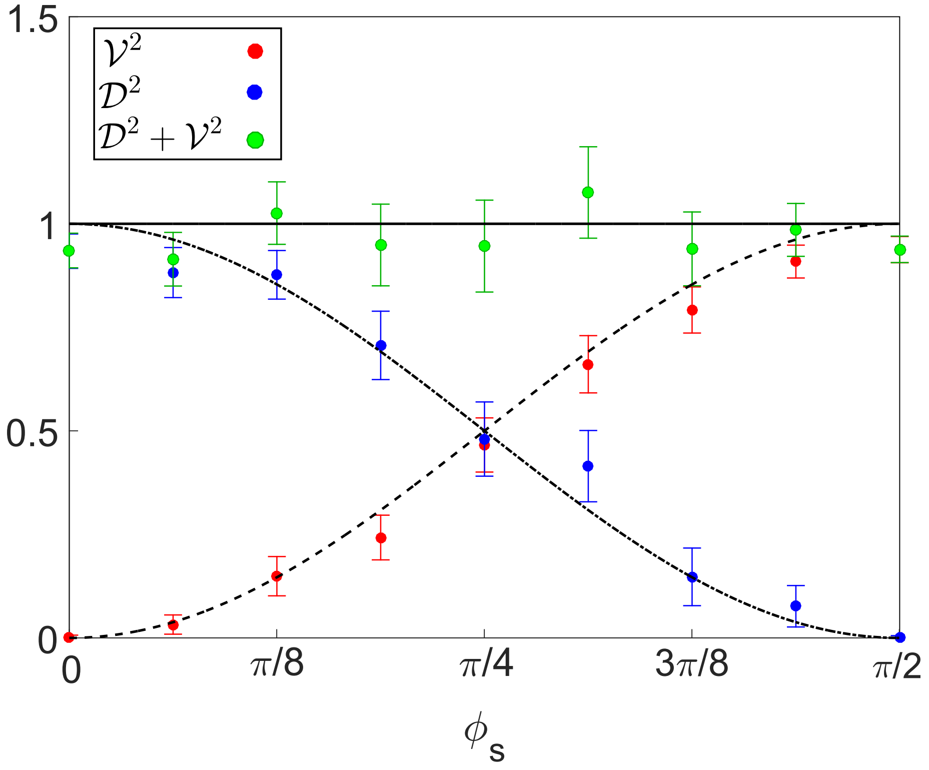

Fig. S1. Wave-particle duality. We plot the visibility and distinguishability of OAM states in our experimental setup for different values of the TBS ($\boldsymbol{\phi_s}$). We also plot the which-way inequality [Eq. (S1)] by summing the visibility and distinguishability squares, showing that we are satisfying it within the error bars. Error bars were calculated using error propagation assuming Poissonian statistics for the recorded number of detection events.
